# Implementation of an IT-guided checklist to improve the quality of medication history records at hospital admission

**DOI:** 10.1007/s11096-017-0545-0

**Published:** 2017-10-29

**Authors:** Tanja Huber, Franziska Brinkmann, Silke Lim, Christoph Schröder, Daniel Johannes Stekhoven, Walter Richard Marti, Richard Robert Egger

**Affiliations:** 1 Hospital Pharmacy, Cantonal Hospital Aarau, Aarau, Switzerland; 2 Department of Surgery, Cantonal Hospital Aarau, Aarau, Switzerland; 3 Statistical Consulting, Quantik, Berikon, Switzerland

**Keywords:** Hospital, Medication discrepancies, Medication history, Patient safety, Quality improvement, Switzerland

## Abstract

*Background* Medication discrepancies often occur at transition of care such as hospital admission and discharge. Obtaining a complete and accurate medication history on admission is essential as further treatment is based on it. *Objective* The goal of this study was to reduce the proportion of patients with at least one medication discrepancy in the medication history at admission by implementing an IT-guided checklist. *Setting* Surgery ward focused on vascular and visceral surgery at a Swiss Cantonal Hospital. *Method* The study was divided into two phases, before and after implementation of an IT-guided checklist. For both phases a pharmacist collected and compared the medication history (defined as gold standard) with that of the admitting physician. Medication discrepancies were subdivided in omissions and commissions, incorrect medications or dose changes, and incorrect dosage forms or strength. *Main outcome measure* The proportion of patients with at least one medication discrepancy in the medication history before and after intervention was assessed. *Results* Out of 415 admissions, 228 patients that met the inclusion criteria were enrolled in the study, 113 before and 115 patients after intervention. After intervention, medication discrepancies declined from 69.9 to 29.6% (*p* < 0.0001) of patients, the mean medication discrepancy per patient was reduced from 2.3 to 0.6 (*p* < 0.0001), and the most common error, omission of a regularly used medication, was reduced from 76.4 to 44.1% (*p* < 0.001). *Conclusion* The implementation of the IT-guided checklist is associated with a significant reduction of medication discrepancies at admission and potentially improves the medication safety for the patient.

## Impact on practice

- Systematic acquisition of medication history at hospital admission potentially improves medication safety.
- IT-guided checklists have a great potential for an accurate medication history at the time of hospital admission.

## Introduction

Approximately one quarter of all admissions in an acute care hospital are related to adverse drug events [1, 2]. Transition of care events such as admission, transfer within the hospital, and discharge from the hospital contribute greatly to adverse drug events that are associated with prolonged hospital stay, added costs, and increased mortality [3, 4]. Often, adverse drug events are the result of medication discrepancies, that are unintended documentation errors at transition of care [4].

An initial source of potential medication errors lies within the proper acquisition of the patient’s medication history [5, 6]. A systematic review of 22 studies uncovered that between 10 and 67% of patients had at least one medication discrepancy at hospital admission [7]. In 6 of these studies, up to 59% of the found discrepancies were considered to be clinically important [7]. The most common error in the medication history was the omission of a regularly taken medication [5, 6, 8, 9].

The inaccurate or incomplete recording of the medication history at admission can lead to consequences such as interrupted or inappropriate drug therapies, duplications, or unforeseen drug interactions with an increased risk for drug-related problems that may not only persist during hospital stay but continue after discharge [3, 6, 10, 11].

Medication reconciliation interventions were previously shown to reduce discrepancies in the medication history that may lead to complications and subsequently prolonged hospital stays [12–14].

## Aim of the study

The aim of the study was to reduce the proportion of patients with at least one medication discrepancy in the medication history at hospital admission by implementing an in-house developed electronic checklist.

## Ethics approval

The study was approved by the National Swiss Ethics Committee on research involving humans (#2016-00939).

## Method

### Study design

**Fig. 1:**
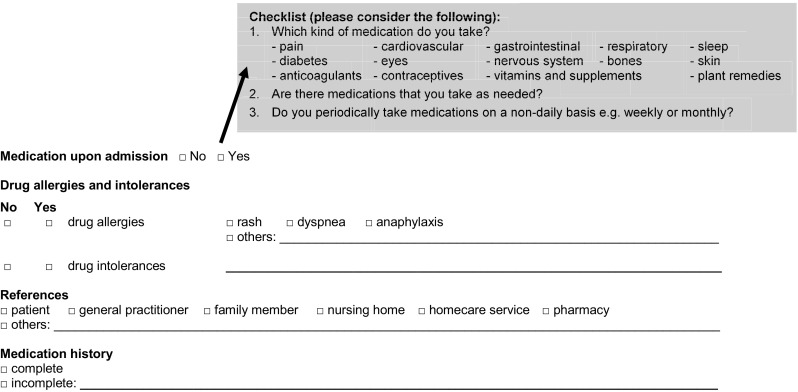
Checklist used in the electronic prescribing system (EPS) for this study (translated version)

The prospective study was conducted in a ward with 26 beds focusing on vascular and visceral surgery at the Kantonsspital Aarau, a Swiss cantonal hospital. To support physicians in obtaining accurate and complete medication histories of patients at admission, a multidisciplinary team of pharmacists, physicians and nurses developed a checklist in the electronic prescribing system (EPS) to provide a framework and guide during the acquisition of the necessary information (Fig. 1).

Admitted patients were enrolled during the two phases of the study. Phase 1, before the introduction of the checklist, lasted from November 2013 to January 2014. The effect of the checklist on medication history accuracy was examined during phase 2, which lasted from February 2015 to May 2015. Before implementing the change to using the checklist, all involved surgeons had to attend a mandatory seminar on how to use the new tool.

During both phases, the medication history for each patient was acquired by a physician and compared to that prepared by the same pharmacist (gold-standard). All identified medicines were documented according to the ATC code system. Each divergence was considered a medication discrepancy and categorized in six groups:

- Omission of a medication.
- Commission of a medication (additional drug not used before admission).
- Incorrect medication.
- Incorrect strength.
- Incorrect dosage form.
- Incorrect dose change.

The medication management of the patients prior to admission was categorized in five groups:

- A = at home without care service support.
- B1 = at home with nursing support.
- B2 = at home with family member support.
- B3 = at home with pharmacist support.
- I = at nursing home.

Patient admission was subdivided into three groups:

- E = elective admission.
- N = admission through the emergency department.
- SDS = admission for same day surgery.

### Study population

All admitted patients (≥ 16 years), who were transferred to the surgical ward, were included.

Patients, who were discharged from hospital within 48 h or had been moved to another ward, before the pharmacist could obtain the medication history, were all excluded from the study. Likewise, patients, who were admitted for same day surgery in phase 2 or with communication problems and without a family member to be interviewed, were not included in the study.

### Data collection

To collect the medication data of a patient, the pharmacist used a standard structured questionnaire. When necessary, information was assembled from the general practitioner, the patient’s pharmacy, nursing home or a family member. To ensure that the physicians had adequate time to collect the medication history, the two medication lists were compared two working days after the patient’s arrival at the ward, and all medication discrepancies were documented.

### Statistics

Before starting to collect data, a power analysis was conducted using the software R-2.15.2 (2012-10-26, Trick or Treat). Using a Poisson regression (α = 0.05 and β = 0.05) and assuming the intervention would reduce the proportion of patients with at least one medication discrepancy by 50%, a minimum enrollment of 110 patients per phase was needed. Microsoft Excel 2010 and R-3.1.3 (2015-03-09, Smooth Sidewalk) were used for the descriptive and statistical analysis. Significance level for adjusted *p* values was set to 0.05. In order to check whether the patient group in phase 1 differed from phase 2 univariate tests were performed. Exact Fisher’s tests were used for nominal variables, while Welch t-tests were performed for continuous variables. Potential differences between medication discrepancies in the two phases were assessed using an Exact Fisher’s test. *P* values were corrected for multiple testing using the method of Benjamini and Hochberg. Exact Fisher’s tests were further used to calculate the odds ratios (OR) for using the checklist and obtaining a systematic medication history. Influence of other factors was analyzed using logistic regression. The suitability of the model was assed using graphical diagnostics of the fit and distributional assumptions.

## Results

228 of 415 patients admitted to the ward during the two phases were included in this study. 113 were included in phase 1 before intervention and 115 in phase 2 after intervention. 187 patients were excluded for the following reasons: 125 were discharged within less than 48 h, 39 were admitted for same day surgery during phase 2, 15 moved to another ward before a pharmaceutical interview could be conducted, 7 due to communication issues, and 1 for refusing to be interviewed.

The characteristics of the patients during phase 1 and phase 2 are shown in Table 1. 86% were admitted during daytime (93.8% in phase 1, 78.3% in phase 2) and 60.1% (54.0 and 66.1%, respectively) due to emergencies (N). While 14.2% were admitted for same day surgery (SDS) in phase 1, no SDS admissions were included in phase 2. The largest groups underwent vascular surgery (47.4%) or visceral surgery (37.3%). There were slightly more men than women in phase 1 (58%) and substantially more in phase 2 (85%). There were similar types of care services used by the patients recruited during phase 1 and 2.

**Table 1:** Characteristics of the patient populations in phase 1 and phase 2

Variable	Levels	n_*before*_	%_before_	n_*after*_	%_*after*_	n_*all*_	%_*all*_
Gender	Male	58	51.3	85	73.9	143	62.7
	Female	55	48.7	30	26.1	85	37.3
*p* = 0.00058	All	113	100.0	115	100.0	228	100.0
Type of admission	E	36	31.9	39	33.9	75	32.9
	N	61	54.0	76	66.1	137	60.1
	SDS	16	14.2	0	0.0	16	7.0
*p* = 0.0005	All	113	100.0	115	100.0	228	100.0
Time of admission	Daytime, 6 a.m.–6 p.m.	106	93.8	90	78.3	196	86.0
	Nighttime, 6 p.m.–6 a.m.	7	6.2	25	21.7	32	14.0
*p* = 0.00096	All	113	100.0	115	100.0	228	100.0
Discipline	a	1	0.9	2	1.7	3	1.3
	g	45	39.8	63	54.8	108	47.4
	tr	13	11.5	8	7.0	21	9.2
	tx	5	4.4	6	5.2	11	4.8
	v	49	43.4	36	31.3	85	37.3
*p* = 0.14	All	113	100.0	115	100.0	228	100.0
Type of case service	A	93	82.3	91	79.1	184	80.7
	B1	3	2.6	3	2.6	6	2.6
	B2	11	9.7	15	13.0	26	11.4
	B3	2	1.8	1	0.9	3	1.3
	I	4	3.5	5	4.3	9	4.0
*p* = 0.91	All	113	100.0	115	100.0	228	100.0

The numbers (n) and the percentage (%) of the included patients for each category are shown. Type of admission: (E) elective, (N) emergency, (SDS) same day surgery. Type of surgeries: (g) vascular, (v) visceral, (tr) trauma, (tx) thoracic, (a) unspecified surgeries. Type of care service: (A) at home without care service support, (B1) at home with nursing support, (B2) at home with family member support, (B3) at home with pharmacist support, (I) at nursing home

The descriptive summary of continuous variables is summarized in Table 2. The median age of the patients in both phases was 67 years (IQR 52-78). The median number of regularly used medication was 5 (IQR 2-10), and the number of discrepancies in the medication history was reduced from a maximum of 14 down to 8 after intervention (*p* < 0.0001). All other variables showed no difference.

**Table 2:** Descriptive summary of the continuous variables in the data

Variable	Levels	n	Min	q1	$\tilde{x}$	$\bar{x}$	q3	Max	s	IQR	#NA
Age	Before	113	16	51.0	67	64.2	78.0	95	18.0	27.0	0
	After	115	19	52.5	67	62.7	77.0	91	18.4	24.5	0
*p* = 0.54	All	228	16	51.8	67	63.5	78.0	95	18.2	26.2	0
Medication at admission	Before	113	0	2.0	6	6.5	9.0	22	5.1	7.0	0
	After	115	0	1.0	5	5.9	10.0	20	4.9	9.0	0
*p* = 0.35	All	228	0	2.0	5	6.2	10.0	22	5.0	8.0	0
Hospital stay (day)	Before	113	3	6.0	7	11.2	12.0	66	10.9	6.0	0
	After	114	3	6.0	8	12.9	17.0	63	10.6	11.0	1
*p* = 0.25	All	227	3	6.0	8	12.0	15.0	66	10.8	9.0	0
Numbers of medication discrepancies	Before	113	0	0.0	2	2.3	3.0	14	2.6	3.0	0
	After	115	0	0.0	0	0.6	1.0	8	1.3	1.0	0
*p* < 0.0001	All	228	0	0.0	0	1.4	2.0	14	2.2	2.0	0

For each variable the number of observations (n), the minimum (Min), the first quartile (q1), the median ($\tilde{x}$), the mean ($\bar{x}$), the third quartile (q3), the maximum (Max), the standard deviation (s) and the inter-quartile range (IQR) are given. For missing data see column #NA (not available)

**Fig. 2:**
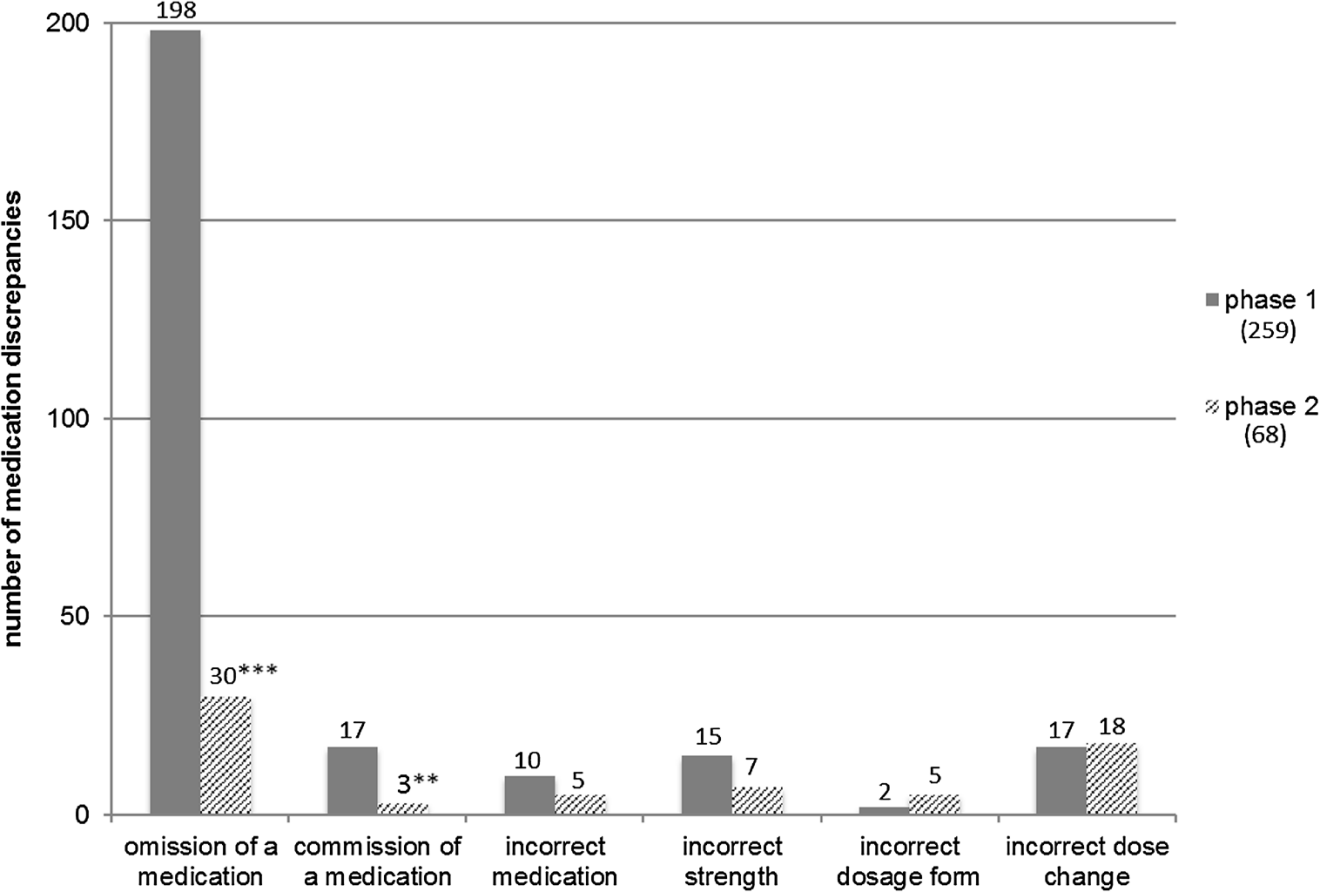
Medication discrepancies for different types of errors before and after intervention. Statistical significance before and after intervention is indicated by asterisks: *** *p* < 0.001; ** *p* < 0.01. There is no significant difference in the columns without asterisks

Out of 735 medications checked before intervention, 259 (35.2%) contained discrepancies (Fig. 2). 69.9% of the patients had at least one medication discrepancy. The most common discrepancy in phase 1 was omission of a regularly used medication (76.4%) followed by the commission of a medication (6.6%). After intervention, the discrepancies were dramatically reduced to 68 out of 677 checked medications (10%) with a significant decrease in medication omissions down to 44.1% (*p* < 0.001) and commission to 4.4% (*p* < 0.01). Only 29.6% of patients had at least one discrepancy after intervention (*p* < 0.0001), and the rate per patient decreased from 2.3 to 0.6 (Table 2). With the IT-guided checklist the odds ratio of having a medication discrepancy was 4.9 times lower (OR 4.9, 95% confidence interval 3.6–6.6).

The most common discrepancy in both phases was omission of a medication. In phase 1, the most discrepancies occurred in the group of analgesics (ATC code N02) at 17.2% leading ophthalmologicals (S01) at 7.6% and mineral supplements (A12) at 7.1%. After the implementation of the IT-guided checklist, the medication discrepancies for analgesics were reduced from 34 to 4, for ophthalmologicals from 15 to 8 and for mineral supplements from 14 to 1.

Using logistic regression, various factors were examined for their influence on the OR (Table 3). There were two significant parameters. On one hand, the odds for a medication discrepancy were reduced by 74% by using the checklist. On the other hand, there was a 1.7% decrease in the odds of having a medication discrepancy for each additional year of age (*p* = 0.015).

**Table 3:** Multiple logistic regression analysis of potentially influential factors

Factors	Coefficient	Adjusted *p* value
(Intercept)	0.108	0.929
Female	0.300	0.176
Admission through the emergency department	0.324	0.198
Admission for same day surgery	0.048	0.929
Nighttime admission	−0.297	0.547
Age	−0.017	**0.015**
Vascular surgery	−0.289	0.929
Traumatology	0.054	0.929
Thoracic surgery	0.108	0.929
Visceral surgery	0.263	0.929
At home with nursing support	0.317	0.534
At home with family member support	−0.424	0.176
At home with pharmacist support	−0.056	0.929
At nursing home	−0.108	0.929
Hospital stay	0.014	0.123
IT-guided checklist	−1.361	**0.000**

Significance level for adjusted* p* values was set to 0.05

The reference parameters for the Intercept are the male gender, elective admission, daytime admission, unspecified surgeries and at home without care service

Age was compared to the type of care service to see if there is an association between these two factors. The combination showed a slight trend but no statistical significance.

## Discussion

The acquisition of the medication history at admission was found to be a major source for medication discrepancies [5, 6, 8, 9]. By implementing an IT-guided checklist, our findings show comparable results to other intervention studies [12, 14], indicating that medication discrepancies can be reduced significantly by reconciliation. One study achieved a decrease of postoperative medication discrepancies from 40.2% of the patients in the standard care arm to 20.3% in the intervention arm [12]. Another study reduced the number of clinically relevant medication discrepancies per patient with a web-based intervention from 1.44 to 1.05 [14]. In this study, the mean number of discrepancies per patient was reduced from 2.3 to 0.6 (Table 2). A possible explanation for differences between the studies could be found in how a medication discrepancy is defined. The medication discrepancies in our study were not classified based on their potential to cause harm. We also did not restricted age or the number of medications at admission like other studies [5, 6, 8]. Therefore, it is not possible to holistically compare our results with previous studies.

Our results revealed that by far the most common discrepancy in both phases was omission of a medication. Our finding of 76.4% omissions before intervention is higher if compared to previous studies (46.6–62%) [5–9, 12]. As mentioned above, the difference could be found in differences in methods, concepts and in the analysis of the outcomes. Nevertheless, the implementation of the IT-guided checklist reduced the incidence of omission significantly to 44.1%.

Among the omitted medication, analgesics were the most frequently forgotten drug classes in the medication history. This result is consistent with a study showing that analgesics are one of three categories of drugs that cause most discrepancies [9]. Also often missing were ophthalmologicals and mineral supplements. A possible explanation for mineral supplements is provided by Cockayne et al. [15]. The study showed that complementary and alternative medicines, so called CAM, are rarely documented [15]. We speculate that drug classes like ophthalmologicals and mineral supplements are not always perceived as medication by patients. Thus, it is necessary to specifically address the usage of these drug classes with the patient. Since our checklist reminded the physician to specifically address these types of medication, it is not surprising that the implementation of the checklist significantly reduced the medication discrepancies of these drug classes.

The second most common discrepancy before intervention was commission of a medication (6.6%). One possibility that can lead to commission is copying from previous medication lists. A previous study has found that out of 120 patients 69.2% had one medication list, 26.0% had two, 4.0% had three and 0.8% had four lists [16]. We also encountered patients, who had several medication lists that differed from each other. Since the frequency of such incidents was not documented in phase 1 or phase 2, we cannot assess, how much they contributed to commission of a medication and the decrease of such incidences from 6.6 to 4.4% after intervention.

The finding that a medication list does not guarantee that a patient actually takes all the medication on the list underlines the importance that a medication list should not just be copied but used in combination with information from other sources, most importantly from an interview with the patient [16, 17]. The concept of medication reconciliation is that cross-referencing information from as many sources as possible will lead to a more complete and accurate medication history [18]. The goal of this study was to provide the physicians with a structured approach on how to obtain the most accurate and complete medication history. Thus, we can assume that the reductions in omission and commission were largely due to the implementation of the IT-guided checklist.

The numbers for other types of discrepancies before and after intervention such as incorrect medication, incorrect strength, incorrect dosage form and incorrect dose change did not change significantly (Fig. 2). The discrepancy-rate for incorrect medication and incorrect strength was reduced, but the numbers were too small to be significant. It is possible that a significant effect could be seen with a larger set of data.

The two study groups of phase 1 and 2 differed in some aspects. For instance, the proportion of male patients and of patients who were admitted during the night was somewhat higher during phase 2 (Table 1). Although there was an increase in patients for same day surgery in phase 2, they were excluded since the attending anesthetists did not use the checklist to record the medication history. In general, large differences between two study groups are not desirable, since negatively affect the comparison. In this study, minor differences between the study groups did not appear to affect the overall reduction of medication discrepancies when using the checklist (*p* < 0.0001). In addition to the checklist, increasing age was found to significantly reduce the number of discrepancies. This was surprising, because age has been shown in the literature to be a potential risk factor [19–21]. However, another study has found that patients 85 years and older have a lower risk for potentially harmful medication errors [22]. A possible explanation for age as a protective factor could be that patients of increased age, who are either admitted from nursing homes or have some kind of homecare service, are admitted with a more complete and accurate list of their medication. Our data show a slight trend for such a connection but no statistical significance. Further investigations are necessary to examine this hypothesis.

The study has several limitations. It was conducted with patients of only one surgical ward and in only one hospital, where admission process may be different from other hospitals. A further limitation is that the obtained data from the same day surgery patients were not excluded from phase 1. At the end of phase 1 it was not clear that the anesthetists would not be utilizing the checklist in phase 2. By retrospectively excluding the same day surgery patients form phase 1, the required sample size of 110 patients would not have been met.

As mentioned earlier, the discrepancies were not classified based on their potential to cause harm. Further research is needed in order to determine the impact on medication safety by the implemented IT-guided checklist.

Our regression model was defined as the discrepancies divided by the number of used medications. We would like to point out that the number of discrepancies could exceed the number of medications per patient, since a single medication can provoke multiple discrepancies like incorrect strength and incorrect dose change. There was one case, where more discrepancies than regularly used medications were made. In this case, we set the number of correct medication to 0. It can also not be ruled out that some medication discrepancies remained undetected, since the same pharmacist recorded the medication history and identified the discrepancies. However, these limitations appear to only marginally affect the apparent result of significantly reducing medication discrepancies by using a checklist at admission.

## Conclusions

Discrepancies in the medication history were found to be significantly reduced by providing a standardized, IT-guided checklist to the physicians. Since the lack of a complete medication history leaves patients at risk for medication errors, the intervention applied in this study will likely enhance the medication safety and reduce complications due to drug-related problems.
